# Compressive strength prediction and low-carbon optimization of fly ash geopolymer concrete based on big data and ensemble learning

**DOI:** 10.1371/journal.pone.0310422

**Published:** 2024-09-12

**Authors:** Peiling Jiang, Diansheng Zhao, Cheng Jin, Shan Ye, Chenchen Luan, Rana Faisal Tufail

**Affiliations:** 1 Zhejiang Tongji Vocational College of Science and Technology, Zhejiang, China; 2 Zhejiang University of Technology, Zhejiang, China; 3 Zhejiang University of Technology Engineering Design Group Co. Ltd, Zhejiang, China; 4 Institute of Advanced Study, Chengdu University, Chengdu, China; 5 School of Civil and Environmental Engineering, Harbin Institute of Technology, Shenzhen, China; 6 Civil Engineering Department, Wah Campus, COMSATS University Islamabad, Rawalpindi, Pakistan; Jazan University, SAUDI ARABIA

## Abstract

Portland cement concrete (PCC) is a major contributor to human-made CO_2_ emissions. To address this environmental impact, fly ash geopolymer concrete (FAGC) has emerged as a promising low-carbon alternative. This study establishes a robust compressive strength prediction model for FAGC and develops an optimal mixture design method to achieve target compressive strength with minimal CO_2_ emissions. To develop robust prediction models, comprehensive factors, including fly ash characteristics, mixture proportions, curing parameters, and specimen types, are considered, a large dataset comprising 1136 observations is created, and polynomial regression, genetic programming, and ensemble learning are employed. The ensemble learning model shows superior accuracy and generalization ability with an RMSE value of 1.81 MPa and an R^2^ value of 0.93 in the experimental validation set. Then, the study integrates the developed strength model with a life cycle assessment-based CO_2_ emissions model, formulating an optimal FAGC mixture design program. A case study validates the effectiveness of this program, demonstrating a 16.7% reduction in CO_2_ emissions for FAGC with a compressive strength of 50 MPa compared to traditional trial-and-error design. Moreover, compared to PCC, the developed FAGC achieves a substantial 60.3% reduction in CO_2_ emissions. This work provides engineers with tools for compressive strength prediction and low carbon optimization of FAGC, enabling rapid and highly accurate design of concrete with lower CO_2_ emissions and greater sustainability.

## 1. Introduction

### 1.1. Background

Portland cement is the essential component of concrete, the most consumed material in the world after water; However, it accounts for 8% of human-made CO_2_ emissions, contributing significantly to climate change [1, 2]. To address this, geopolymer, also known as low-calcium alkali-activated material, has gained worldwide attention as a promising low-carbon substitute for cement [1, 2]. It is synthesized using industrial by-products or waste ashes containing glassy/amorphous Si and Al with a small alkali-activator. Fly ash, a prevalent by-product of coal combustion, is an ideal precursor for the geopolymer. It is an excellent source of amorphous SiO_2_ and Al_2_O_3_. Meanwhile, it is also the largest industrial by-product in the world. With global coal combustion generating one billion tons of fly ash annually, of which only 30% is utilized [3], the potential for fly ash geopolymer concrete (FAGC) to mitigate CO_2_ emissions is substantial. Compared with conventional Portland cement concrete (PCC), FAGC not only reduces CO_2_ emissions [4, 5] but also exhibits comparable engineering properties [6, 7], better durability in sulfates or acids [8], and higher fire resistance [9, 10].

A key issue for concrete, including but not limited to FAGC, is the mixture design optimization, i.e., determining the optimal mixture proportions to yield a concrete that meets performance targets, such as compressive strength, while minimizing CO_2_ emissions [11, 12]. In response to the challenges posed by climate change, the focus of concrete research today has shifted from enhancing concrete performance to reducing its environmental impact while ensuring performance [13]. While traditional trial batching (experimental design) is one approach to mixture design optimization, it involves conducting numerous trials with various mixture parameters as variables. As the number of variables increases, the required number of trials grows exponentially, making the process time-consuming and resource-intensive. Additionally, this approach only gives relatively well-performing, rather than truly optimal, concrete mixtures.

To overcome the limitations of experimental design, modeling the relationships between concrete properties (such as compressive strength) and mixture proportions enables computational design. This mathematical approach can save time, labor, and resources, and potentially identify the truly optimal mixture proportions [11, 12, 14]. However, due to the highly nonlinear and complex nature of the relationships between concrete properties and mixture proportions, they cannot be captured by simple equations. In such cases, machine learning techniques offer valuable solutions to model complex relationships, provided a representative database of experimental results is available. Detailed and in-depth state-of-the-art reports on the engineering application of machine learning techniques are well-documented in the literature [15–18]. The successful use of machine learning in modeling cement concrete properties has also been extensively covered [19–22]. Furthermore, FAGC, as a highly promising low-carbon concrete, has attracted increasing interest in developing compressive strength prediction models using machine learning techniques [23–25].

**Fig 1:**
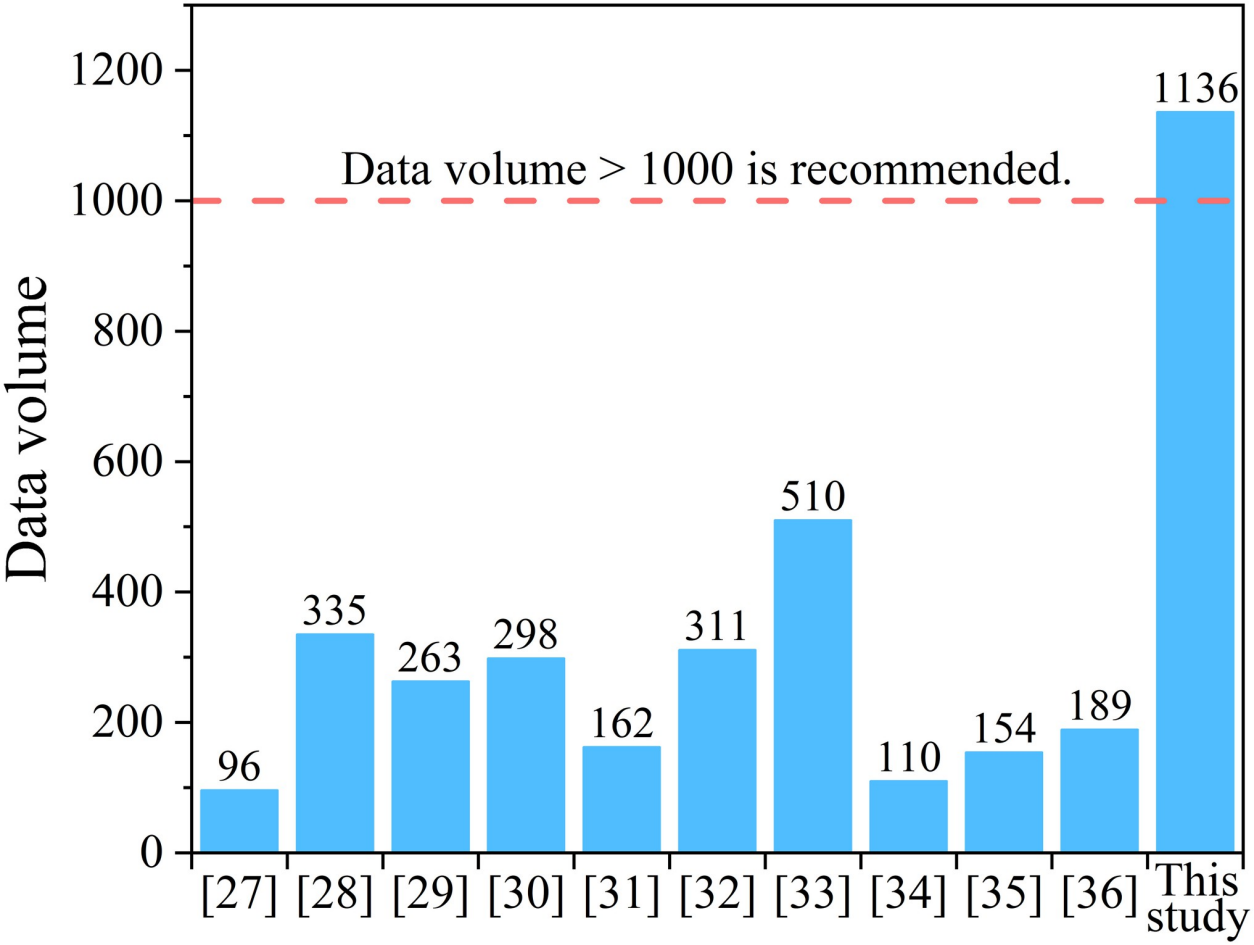
Comparison of data volume in different studies.

Despite the advantage of machine learning in modeling complex relationships, the risk of overfitting remains a significant challenge [21, 26]. Overfitting means that although a model performs exceptionally well on the training data, the model gives inaccurate predictions for new data. Preventing overfitting in machine learning is crucial, and several strategies exist to address this issue. One effective strategy is to increase the size of the training dataset. A dataset exceeding 1000 observations is recommended [18]. However, most prior studies [27–36] on modeling the compressive strength of FAGC are limited to small data sets with 100–500 observations, as shown in Fig 1. Another approach to reducing overfitting risk is the use of ensemble learning methods, such as stacking. Compared with the use of a single machine learning model, stacking ensemble learning combines the prediction results of multiple models through weighting or other methods to produce a final prediction, usually achieving higher accuracy and better generalization ability, i.e., better predictive ability for unknown data. Asteris et al. [26] were the first to apply stacking ensemble learning to predict the compressive strength of concrete materials. They used four conventional machine learning techniques and combined the outputs using an artificial neural network to create the stacking ensemble model, which outperformed the individual models. Later, Li and Song [37] applied a similar approach to predict the compressive strength of PCC with rice husk ash, developing decision tree and extreme gradient boosting models and then combining outputs using linear regression. Their stacking ensemble model achieved higher accuracy and better performance on new datasets compared to single models.

In summary, despite the progress made in developing compressive strength prediction models for FAGC based on mixture proportions, there is currently a lack of studies that utilize large datasets and ensemble learning methods. Moreover, while compressive strength prediction models are essential, they are only part of the broader objective of mixture design optimization. A tool for mixture design optimization of FAGC is still lacking. Besides compressive strength prediction models, mixture design optimization also requires modeling the relationship between CO_2_ emissions and mixture proportions and solving the concrete mixture design optimization problem [11, 12].

Considering these points, this study leverages a dataset comprising 1136 observations and employs an ensemble machine learning method to develop a robust compressive strength prediction model for FAGC. Then, based on the strength model and life cycle assessment (LCA)-based CO_2_ emissions model, this study develops an optimal FAGC mixture design program to design the FAGC mixture that satisfies the target strength and minimizes CO_2_ emissions. Finally, a case study is presented to evaluate the effectiveness of the optimal mixture design tool.

### 1.2. Research significance and novelty

To address the global challenges of climate change, FAGC has been widely considered a promising low-carbon substitute for conventional concrete. This study addresses the critical need for computational mixture design optimization to develop FAGC that meets key performance targets, such as compressive strength, while minimizing CO_2_ emissions. Achieving this goal requires a compressive strength prediction model with good generalization ability. Despite many studies on developing related models, there is currently a lack of studies using large datasets and ensemble learning methods—two crucial strategies for enhancing generalization. This study addresses this gap by using a dataset comprising 1136 observations and employing an ensemble machine learning method to develop a compressive strength prediction model for FAGC. To the author’s best knowledge, the database reported in this paper is the largest to date. The model exhibits superior accuracy and generalization ability in testing and validation datasets. Beyond merely predicting compressive strength, this study integrates this strength model and an LCA-based CO_2_ emissions model and develops a mixture design optimization tool to design the FAGC mixture that satisfies the target strength and minimizes CO_2_ emissions. The results of this work provide engineers with tools for compressive strength prediction and low-carbon optimization of FAGC, enabling rapid and highly accurate design of concrete with lower CO_2_ emissions and greater sustainability.

## 2. Materials and methods

### 2.1. Research process

**Fig 2:**
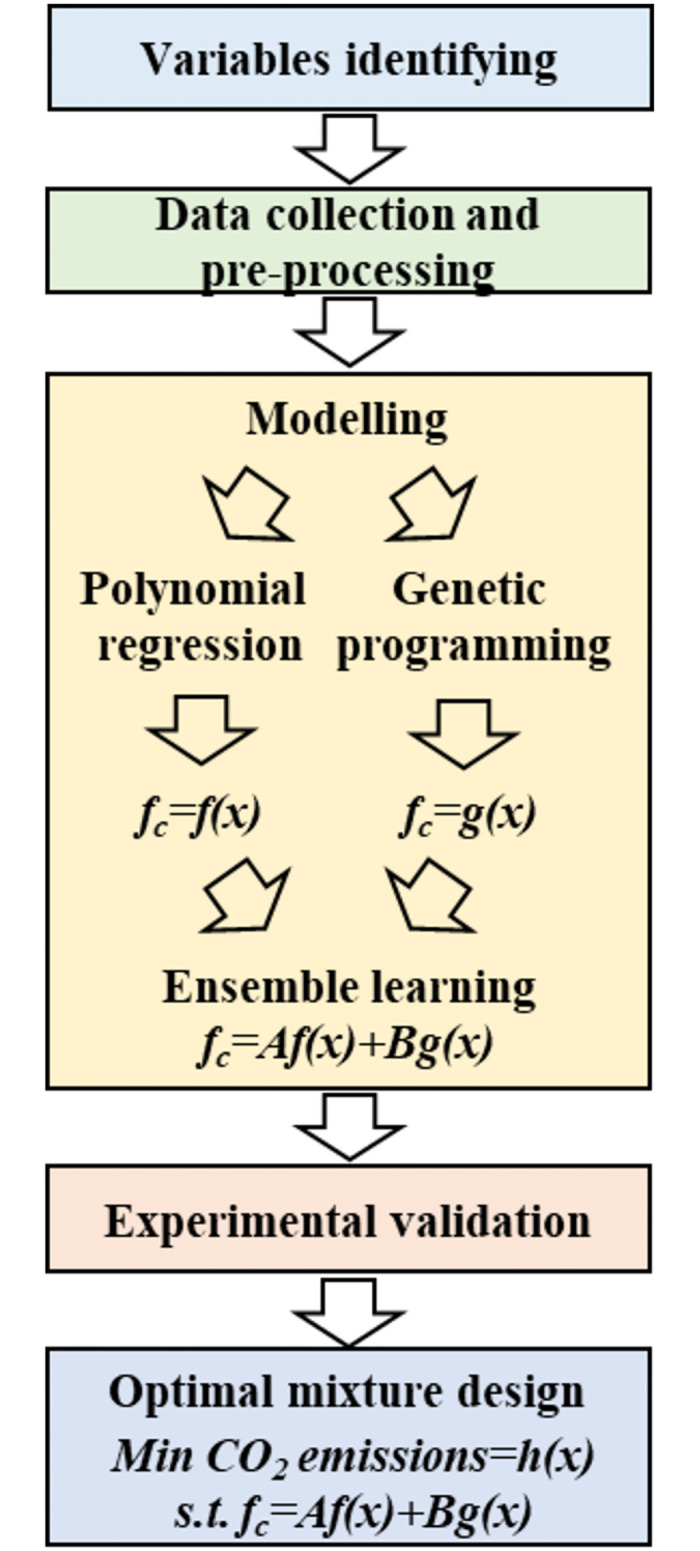
Research process flowchart.

Fig 2 illustrates the research process flowchart in this study, encompassing variable identification, data collection and preprocessing, modeling, experimental validation, and optimal mixture design. The study begins by identifying the input variables for modeling (Section 2.2). Subsequently, only data that provides information on all identified input variables is collected from the literature, resulting in a big dataset with 1136 observations (Section 2.3). Data preprocessing is a critical step, as it directly affects the performance and accuracy of the models (Section 2.4). Polynomial regression and genetic programming are employed to develop the prediction model, with outputs combined using linear regression to form the stacking ensemble model (Section 2.5). The dataset is randomly split into training and testing sets, with 75% of the data used for training, and the remaining 25% reserved for testing the models’ performance. Additionally, new experimental data is used to further assess the proposed models (Section 2.6). A comparison of the proposed models is conducted using performance indices (Section 2.7), with the best-performing model selected. Finally, an optimal mixture design method is developed, aiming to achieve target compressive strength while minimizing CO_2_ emissions, based on the strength model and an LCA-based CO_2_ emissions model (Section 2.8). The objective function is to minimize CO_2_ emissions, with achieving the desired compressive strength as a primary constraint, alongside other constraints (Section 2.9). The optimal mixture design is determined by solving the optimization problem defined by the objective function and constraints, using the scipy.optimize.minimize algorithm within Python’s optimization toolbox.

### 2.2. Variable identifying

This section discusses the factors influencing the compressive strength of FAGC and identifies the input variables in modeling. Table 1 compares the influencing factors considered in previous studies. The influencing factors include fly ash characteristics, mixture proportions, curing parameters, and specimen type. Previous studies only considered part of the influencing factors. To develop a robust compressive strength prediction model of FAGC, this study considers the fly ash characteristics, mix proportions, curing parameters, and specimen type together. The detailed parameters considered in this study include SiO_2_, Al_2_O_3_, Fe_2_O_3_, and CaO percent in fly ash (*P*_SiO2,FA_, *P*_Al2O3,FA_, *P*_Fe2O3,FA_, *P*_CaO,FA_), molar ratios of Na to Al (*Na/Al*) and Si to Al (*Si/Al*), mass ratios of H_2_O to fly ash (*H*_*2*_*O/FA*), coarse aggregate to fly ash (*C/FA*), and fine aggregate to fly ash (*F/FA*), heat-curing temperature (*T*), heat-curing time (*t*_*1*_), and total curing time (*t*_*2*_). These parameters cover fly ash characteristics, mixture proportions, and curing parameters. Besides, this study also considers the effect of specimen shape by converting cylinder strength to cube strength before modeling [38].

**Table 1:** Comparison of influencing factors considered in different studies.

	Mix proportions	Fly ash characteristics	Curing parameters	Specimen type
Lokuge et al. (2018) [27]	**√**	**×**	**×**	**×**
Nguyen et al. (2020) [28]	**√**	**×**	**√**	**×**
Huynh et al. (2020) [29]	**√**	**×**	**√**	**×**
Khan et al. (2021) [30]	**√**	**×**	**√**	**√**
Toufigh & Jafari (2021) [31]	**√**	**√**	**√**	**×**
Chu et al. (2021) [32]	**√**	**×**	**√**	**×**
Ahmad et al. (2021) [33]	**√**	**√**	**√**	**×**
Peng & Unluer (2022) [34]	**√**	**√**	**√**	**×**
Ahmad et al. (2022) [35]	**√**	**×**	**×**	**×**
Khalaf et al. (2022) [36]	**√**	**√**	**√**	**×**
**This study**	**√**	**√**	**√**	**√**

#### 2.2.1. Characteristics of fly ash

Fly ash is a by-product and its properties can vary significantly. This variation in the physicochemical properties of fly ash from different sources can lead to substantial differences in the compressive strength for the same mixture proportion [39]. Therefore, it is crucial to consider the characteristics of fly ash in the model. The oxide composition of fly ash, particularly the percentages of SiO_2_, Al_2_O_3_, CaO, and Fe_2_O_3_, plays a crucial role in determining the characteristics of fly ash. These oxides make up approximately 90% of the composition, while the remaining oxides are negligible. The content of CaO and the total content of SiO_2_, Al_2_O_3_, and Fe_2_O_3_ are used to classify fly ash in ASTM C618-19. These oxides have a significant impact on the compressive strength of FAGC. Si and Al play major roles in geopolymer formation. The general formula of geopolymer is Na_m_[–(SiO_2_)_q_–AlO_2_]_m_·wH_2_O (usually abbreviated as N-A-S-H), where q is *Si/Al* (1, 2, or 3) [40, 41]. SiO_2_ and Al_2_O_3_ are dissolved in an alkaline medium and then re-polymerize to form inorganic polymers with a three-dimensional network structure formed by connecting SiO_2_ tetrahedra and AlO_2_^-^ tetrahedra. Recent studies reported that Fe_2_O_3_ participates in geopolymerization like Al_2_O_3_ [42, 43]. The presence of CaO leads to the formation of C-A-S-H, which accelerates the hardening of geopolymers and promotes early high strength at room temperature [44–46]. Based on the above, this study considers the SiO_2_, Al_2_O_3_, Fe_2_O_3_, and CaO percent in fly ash as input variables. Other fly ash characteristics, such as the amorphous content, fineness, and density, also influence the strength. However, as most papers only provide the chemical components of fly ash without further details, this model focuses on the oxide components of fly ash as input variables. It may affect the applicability of the model to a certain extent.

#### 2.2.2. Mixture proportions

FAGC is produced by mixing coarse and fine aggregates, fly ash, and alkali-activated solution. The alkali-activated solution comprises Na_2_O, SiO_2_, and H_2_O, and is usually prepared by mixing NaOH solution and Na_2_SiO_3_ solution (water glass). The water-reducing admixture is not involved because the geopolymer system lacks effective water-reducing admixture [47]. Therefore, the mixture proportions of FAGC involve mass ratios of Na_2_O to fly ash (*Na*_*2*_*O/FA*) and SiO_2_ to fly ash (*SiO*_*2*_*/FA*) as well as *H*_*2*_*O/FA*, *C/FA*, and *F/FA*. This study uses *Na/Al* and *Si/Al* instead of *Na*_*2*_*O/FA* and *SiO*_*2*_*/FA* because the variations of compressive strength with *Na*_*2*_*O/FA* and *SiO*_*2*_*/FA* are essentially induced by the variations of *Na/Al* and *Si/Al*. Na is from the alkali-activated solution. Al is from fly ash. Si is from both the alkali-activated solution and fly ash. The values of these variables are calculated using Eqs (1–8).

$$Na/Al=\left(M_{\text{NaOH}}/40+M_{\text{Waterglass}}\times P_{\text{Na}2\text{O},\text{WG}}/62\times 2\right)/\left(M_{\text{FlyAsh}}\times P_{\text{Al}2\text{O}3,\text{FA}}/102\times 2\right)$$ (1)

$$M_{\text{NaOH}}=M_{\text{NaOHSolution}}\times P_{\text{NaOH,NS}}$$ (2)

$$Si/Al=\left(M_{\text{FlyAsh}}\times P_{\text{SiO}2,\text{FA}}+M_{\text{Waterglass}}\times P_{\text{SiO}2,\text{WG}}\right)/60/\left(M_{\text{FlyAsh}}\times P_{\text{Al}2\text{O}3,\text{FA}}/102\times 2\right)$$ (3)

$$H_{2}O/FA=\left(M_{\text{H}2\text{O},\text{NS}}+M_{\text{H}2\text{O},\text{WG}}+M_{\text{ExtraAddedH2O}}\right)/M_{\text{FlyAsh}}$$ (4)

$$M_{\text{H}2\text{O},\text{NS}}=M_{\text{NaOHSolution}}\times \left(1\text{-}P_{\text{NaOH},\text{NS}}\times 62/80\right)$$ (5)

$$M_{\text{H}2\text{O},\text{WG}}=M_{\text{Waterglass}}\times \left(1\text{-}P_{\text{Na}2\text{O},\text{WG}}\text{-}P_{\text{SiO}2,\text{WG}}\right)$$ (6)

$$C/FA=M_{\text{CoarseAggregate}}/M_{\text{FlyAsh}}$$ (7)

$$F/FA=M_{\text{FineAggregate}}/M_{\text{FlyAsh}}$$ (8)

where *M*_K_ is the mass of K, and *P*_N,K_ is the mass percent of N in K. For example, *M*_Waterglass_ is the mass of water glass, and *P*_Na2O,WG_ is the mass percent of Na_2_O in water glass. 60, 62, 80, and 102 are the relative molecular mass of SiO_2_, Na_2_O, NaOH, and Al_2_O_3_.

#### 2.2.3. Curing parameters

Curing parameters are widely considered key factors affecting the performances of FAGC [23, 24, 48]. Two-stage curing, i.e., heat curing (e.g., 80°C for 24 hours) followed by room temperature curing, is usually used for FAGC. The strength of FAGC develops slowly at room temperature (about 20°C). Achieving sufficient strength requires at least 56 days [49]. Therefore, slightly-elevated temperature curing for a short time is usually employed to accelerate strength development. The curing process can be described using three variables, namely heat-curing temperature (*T*), heat-curing time (*t*_*1*_), and total curing time (*t*_*2*_). Herein, *t*_*1*_ = *t*_*2*_ means that only heat curing is adopted, and *t*_*1*_ = 0 means that only room temperature curing is adopted.

#### 2.2.4. Specimen shape

Specimen shape needs to be considered, while it is ignored in the previous studies. Researchers from various regions use different specimen shapes for concrete compressive strength testing because the regional standards vary. In contrast to Chinese and European standards, which favor concrete cubes, concrete cylinders with a length-to-diameter ratio of 2 are used in the American standard. The compressive strength results of concrete cubes and cylinders produced by the same batch are different. Cubes have higher compressive strength than cylinders. In this study, the effect of specimen shape is taken into account by converting the cylinder strength to cube strength before modeling. To obtain cube compressive strength, the cylinder compressive strength needs to multiply the factor of 1.18 if it is less than 50 MPa; else, it needs to multiply the factor of 1.04 [38].

### 2.3. Data collection

Only data that provides information on all identified input variables is collected from the literature, resulting in a big dataset with 1136 observations for model development. Table 2 displays part of the dataset. There is too much data to display in full. Please refer to S1 Table for all the data. Table 3 shows the statistical parameters of the dataset. The dataset is randomly split into training and testing sets, with 75% (shown in S2 Table) used for training, and the remaining 25% (shown in S3 Table) for testing. The dataset is created as follows: The literature pertaining to the compressive strength of FAGC are retrieved on the Web of Science and Google Scholar. Afterward, only the literature that offers the key information, such as the SiO_2_, Al_2_O_3_, CaO, Fe_2_O_3_ percent in fly ash, the dosage of fly ash, NaOH solution, Na_2_SiO_3_ solution, extra added H_2_O, coarse aggregate and fine aggregate, the concentration or mass fraction of NaOH solution, the Na_2_O and SiO_2_ content in Na_2_SiO_3_ solution, heat-curing temperature (*T*), heat-curing time (*t*_*1*_), total curing time (*t*_*2*_), the specimen type for strength test, and the compressive strength (*f*_c_), are collected [28, 45, 50–129]. Finally, the key information is transcribed into a spreadsheet.

**Table 2:** Dataset.

No.	*P*_SiO2,FA_ (%)	*P*_Al2O3,FA_ (%)	*P*_Fe2O3,FA_ (%)	*P*_CaO,FA_ (%)	*Na/Al*	*Si/Al*	*H*_*2*_ *O/FA*	*C/FA*	*F/FA*	*T* (°C)	*t*_*1*_ (day)	*t*_*2*_ (day)	*Shape*	*f*_*c*_ (MPa)
1	47.9	28	14.1	3.8	0.47	1.71	0.48	2.55	1.29	80	1	7	Cube	26.6
2	47.9	28	14.1	3.8	0.50	1.79	0.31	2.82	1.43	80	1	7	Cube	39.5
3	47.9	28	14.1	3.8	0.44	1.77	0.30	2.83	1.44	80	1	7	Cube	45.4
4	47.9	28	14.1	3.8	0.48	1.81	0.31	2.82	1.43	80	1	7	Cube	50.6
5	47.9	28	14.1	3.8	0.47	1.71	0.48	2.55	1.29	80	1	28	Cube	28.3
6	47.9	28	14.1	3.8	0.50	1.79	0.31	2.82	1.43	80	1	28	Cube	42.1
7	47.9	28	14.1	3.8	0.44	1.77	0.30	2.83	1.44	80	1	28	Cube	49
8	47.9	28	14.1	3.8	0.48	1.81	0.31	2.82	1.43	80	1	28	Cube	55.5
9	50.2	26.4	10	4.3	0.42	1.86	0.21	2.72	1.81	75	2	7	Cylinder	60.7
10	50.2	26.4	10	4.3	0.42	1.86	0.21	2.72	1.81	75	2	7	Cylinder	59.5
11	50.2	26.4	10	4.3	0.42	1.86	0.21	2.72	1.81	75	2	7	Cylinder	38.5
12	50.2	26.4	10	4.3	0.42	1.86	0.27	2.72	1.81	75	2	7	Cylinder	56.7
13	51.5	23.63	15.3	1.74	0.42	2.12	0.22	2.86	1.62	60	1	28	Cylinder	37
14	51.5	23.63	15.3	1.74	0.46	2.12	0.21	2.86	1.62	60	1	28	Cylinder	64.5
15	51.5	23.63	15.3	1.74	0.50	2.12	0.21	2.86	1.62	60	1	28	Cylinder	72
16	51.5	23.63	15.3	1.74	0.42	2.12	0.22	*2*.*86*	1.62	60	1	28	Cylinder	51
17	51.5	23.63	15.3	1.74	0.42	2.12	0.22	2.86	1.62	60	1	28	Cylinder	52.5
18	51.5	23.63	15.3	1.74	0.42	2.12	0.25	2.94	1.62	60	1	28	Cylinder	26
19	51.5	23.63	15.3	1.74	0.42	2.12	0.25	2.86	1.62	60	1.67	28	Cylinder	33.5
20	32.62	31.23	8.48	17.12	0.36	1.17	0.33	4.57	2.32	20	0	28	Cube	6.93
21	32.62	31.23	8.48	17.12	0.39	1.17	0.32	4.57	2.32	20	0	28	Cube	8.8
22	32.62	31.23	8.48	17.12	0.42	1.17	0.31	4.57	2.32	20	0	28	Cube	13.3
23	32.62	31.23	8.48	17.12	0.48	1.17	0.30	4.57	2.32	20	0	28	Cube	14.9
24	32.62	31.23	8.48	17.12	0.51	1.17	0.30	4.57	2.32	20	0	28	Cube	17.1
25	32.62	31.23	8.48	17.12	0.57	1.35	0.53	4.26	2.16	20	0	28	Cube	8.98
26	32.62	31.23	8.48	17.12	0.63	1.35	0.52	4.26	2.16	20	0	28	Cube	9.55
27	32.62	31.23	8.48	17.12	0.68	1.35	0.51	4.26	2.16	20	0	28	Cube	14.7
~	~	~	~	~	~	~	~	~	~	~	~	~	~	~
1128	66.76	15.48	6.79	5.04	0.56	4.11	0.30	2.61	1.17	20	0	3	Cube	10.1
1129	66.76	15.48	6.79	5.04	0.61	4.11	0.30	2.61	1.17	20	0	3	Cube	13.7
1130	66.76	15.48	6.79	5.04	0.66	4.11	0.29	2.61	1.17	20	0	3	Cube	12.8
1131	66.76	15.48	6.79	5.04	0.56	4.11	0.30	2.61	1.17	20	0	7	Cube	19.8
1132	66.76	15.48	6.79	5.04	0.61	4.11	0.30	2.61	1.17	20	0	7	Cube	20.4
1133	66.76	15.48	6.79	5.04	0.66	4.11	0.29	2.61	1.17	20	0	7	Cube	18.6
1134	66.76	15.48	6.79	5.04	0.56	4.11	0.30	2.61	1.17	20	0	28	Cube	26.5
1135	66.76	15.48	6.79	5.04	0.61	4.11	0.30	2.61	1.17	20	0	28	Cube	29
1136	66.76	15.48	6.79	5.04	0.66	4.11	0.29	2.61	1.17	20	0	28	Cube	27.1

**Table 3:** Statistical parameters of the data set.

	*P*_SiO2,FA_ (%)	*P*_Al2O3,FA_ (%)	*P*_Fe2O3,FA_ (%)	*P*_CaO,FA_ (%)	*Na/Al*	*Si/Al*	*H*_*2*_ *O/FA*	*C/FA*	*F/FA*	*T* (°C)	*t*_*1*_ (day)	*t*_*2*_ (day)	*f*_*c*_ (MPa)
Mean	51.40	24.95	9.23	6.84	0.61	2.25	0.33	2.93	1.58	62.75	0.89	28.45	36.53
Std	11.11	5.87	6.45	7.66	0.28	0.89	0.11	0.72	0.38	24.81	0.68	67.40	14.63
Max	77.2	45.85	40.5	37	2.16	7.76	0.79	6.25	3.11	120	4	540	87.4
Min	17.57	6.37	0.9	0	0.18	0.62	0.15	0.66	0.74	20	0	1	1.1

### 2.4. Data pre-processing

This section presents data pre-processing steps, including normalizing variables and ensuring that the data is in the right distribution. Before modeling, variables should approximate a normal distribution, which can be achieved by taking logarithms. Normalization is also required to scale variables to the same order of magnitude. Table 4 shows the relationship between input variables and model parameters.

**Table 4:** Relationship between variables and model parameters.

Variable	Variable meaning
*x0*	*P*_*SiO2*,*FA*_
*x1*	*P*_*Al2O3*,*FA*_
*x2*	(ln(*P*_*CaO*,*FA*_+0.01)-ln(0.01))/(ln(0.37+0.01)-ln(0.01))
*x3*	(ln(*P*_*Fe2O3*,*FA*_)-ln(0.009))/(ln(0.405)-ln(0.009))
*x4*	(ln(*Na/Al*)-ln(0.1801))/(ln(2.1607)-ln(0.1801))
*x5*	(ln(*Si/Al*)-ln(0.6152))/(ln(7.7583)-ln(0.6152))
*x*6	*H*_*2*_ *O/FA*
*x7*	*C/FA*
*x8*	*F/FA*
*x9*	(*T*-20)/(120–20)
*x10*	*t*_*1*_
*x11*	(ln(*t*_*2*_)-ln(1))/(ln(540)-ln(1))

### 2.5. Modeling techniques

This section presents the basic principles of machine learning techniques used in this work, polynomial regression, genetic programming, and ensemble learning.

#### 2.5.1. Polynomial regression

Based on Taylor expansion, a quadratic function can effectively approximate any nonlinear function. Eq (9) presents an example of the quadratic function involving three input variables, namely *x0*, *x1*, and *x2*. Polynomial regression is essentially multiple linear regression.


$$Y=\theta_{0}+\theta_{1}x0+\theta_{2}x0^{2}+\theta_{3}x0x1+\theta_{4}x0x2+\theta_{5}x1+\theta_{6}x1^{2}+\theta_{7}x1x2+\theta_{8}x2+\theta_{9}x2^{2}$$ (9)

There are 12 input variables in this study. The model initially considers 90 independent variables, namely *x0*, *x0*^2^, *x0x1*, …, *x0x11*, *x1*, *x1*^2^, *x1x2*, …, *x11* and *x11*^2^. However, the correlation between the dependent variable and each selected independent variable is tested using t-test. The independent variable with a P-value of less than 0.05 is considered to have a weak correlation with the dependent variable, and it is deleted. Then, the model is re-solved based on the remaining variables.

#### 2.5.2. Genetic programming

Genetic programming is proposed based on the Theory of Biological Evolution. A formula population is built by randomly combining mathematical operations (+,–, ÷, *(×)), constants, and variables. Then, the fittest individuals are selected based on the fitness (RMSE in this study), and the next generation evolves from them by genetic operations, including crossover, mutation and copy. The iteration continues until the termination condition is met (obtaining a satisfactory formula or exhausting the pre-set iteration number). This study uses an open-source genetic programming “gplearn” based on Python. More detailed information on "gplearn" can be found in its official documentation [130]. It is noted that the division in “gplearn” is specifically defined to avoid dividing by zero. If the dividend *a* is less than 0.001, the result of dividing *b* by *a* is 1. In other cases, the division in “gplearn” is the same as the one we normally use. The goal is to obtain the laws of these factors affecting concrete based on data. The open-source programs provide efficient and effective tools for non-machine learning professionals, such as concrete designers or researchers. They do not need to write machine learning programs themselves but can leverage existing tools to analyze and apply the discovered patterns and laws from machine learning.

#### 2.5.3. Ensemble learning

Firstly, multiple machine learning models are trained. Then, the outputs of the previously trained models are used as inputs to train the ensemble model. In this step, linear regression is commonly used as the training method. For example, Polynomial regression model: *f*_*c*_ *= f(x)*; Genetic programming model: *f*_*c*_ *= g(x)*; ensemble model: *f*_*c*_ *= Af(x)+Bg(x)*. The coefficients *A* and *B* can be determined by linear regression.

### 2.6. Experimental verification

This study uses new experimental data for validating the prediction model of the compressive strength of FAGC. Fly ash from Shenzhen Daote Technology Co., Ltd is used in the experiment, and its oxide composition determined by X-ray fluorescence (PANalytical Axios, the Netherlands) is shown in Table 5. The alkali-activated solution is mixed of sodium silicate solution (Na_2_O = 8.83%; SiO_2_ = 27.64%; H_2_O = 63.53%) with sodium hydroxide (NaOH) solid (Purity > 98%) and tap water, and it is prepared 24 hours in advance before use. River sand with a fineness modulus of 2.5 is used as fine aggregate. Granite crushed stone is used as coarse aggregate. The particle sizes for the coarse aggregate are distributed as follows: 5–10 mm (35%), 10–16 mm (35%), and 16–20 mm (30%), respectively.

**Table 5:** Oxide composition of fly ash (%).

SiO_2_	Al_2_O_3_	CaO	Fe_2_O_3_	P_2_O_5_	Na_2_O	TiO_2_	MgO
57.41	22.82	7.78	6.22	2.22	1.69	0.89	0.67

The mixture proportion is shown in Table 6. A basic scenario (*P*_SiO2,FA_ = 0.5741, *P*_Al2O3,FA_ = 0.2282, *P*_CaO,FA_ = 0.0778, *P*_Fe2O3,FA_ = 0.0622, *Na/Al* = 0.5, *Si/Al* = 2.5, *H*_*2*_*O/FA* = 0.35, *C/FA* = 3, *F/FA* = 1.5, *T* = 80°C, *t*_*1*_ = 1 day, *t*_*2*_ = 28 days) is set, and *Na/Al*, *Si/Al*, *H*_*2*_*O/FA*, *C/FA*, *F/FA*, *T*, *t*_*1*_, and *t*_*2*_ are individually changed to show their effects on the compressive strength of FAGC. The sample preparation and strength testing follow the Chinese standard GB/T 50081–2019 [131], which is equivalent to the European standard EN 12390–3 [132]. The compressive strength is tested on a 2000 KN electro-hydraulic mechanical testing machine (Changchun testing machine factory, Changchun, China), and cubes with a size of 100 mm are used for strength testing.

**Table 6:** Mixture proportion for experimental verification.

*Na/Al*	*Si/Al*	*H*_*2*_ *O/FA*	*C/FA*	*F/FA*	*T* (°C)	*t*_*1*_ (day)	*t*_*2*_ (day)
0.5	2.5	0.35	3	1.5	80	1	28
0.25	2.5	0.35	3	1.5	80	1	28
0.75	2.5	0.35	3	1.5	80	1	28
1	2.5	0.35	3	1.5	80	1	28
1.25	2.5	0.35	3	1.5	80	1	28
0.5	2.25	0.35	3	1.5	80	1	28
0.5	2.75	0.35	3	1.5	80	1	28
0.5	3	0.35	3	1.5	80	1	28
0.5	3.25	0.35	3	1.5	80	1	28
0.5	2.5	0.3	3	1.5	80	1	28
0.5	2.5	0.4	3	1.5	80	1	28
0.5	2.5	0.45	3	1.5	80	1	28
0.5	2.5	0.5	3	1.5	80	1	28
0.5	2.5	0.35	2.7	1.5	80	1	28
0.5	2.5	0.35	3.3	1.5	80	1	28
0.5	2.5	0.35	3	1.2	80	1	28
0.5	2.5	0.35	3	1.8	80	1	28
0.5	2.5	0.35	3	1.5	20	0	28
0.5	2.5	0.35	3	1.5	40	1	28
0.5	2.5	0.35	3	1.5	60	1	28
0.5	2.5	0.35	3	1.5	100	1	28
0.5	2.5	0.35	3	1.5	80	0.25	28
0.5	2.5	0.35	3	1.5	80	0.5	28
0.5	2.5	0.35	3	1.5	80	2	28
0.5	2.5	0.35	3	1.5	80	1	1
0.5	2.5	0.35	3	1.5	80	1	3
0.5	2.5	0.35	3	1.5	80	1	7
0.5	2.5	0.35	3	1.5	80	1	14

### 2.7. Performance indices

The indices for evaluating the model are a root mean squared error (RMSE) computed using Eq (10) and a coefficient of determination (R^2^) computed using Eq (11), which measure the difference between the prediction and experiment. A low value of RMSE and a high value of R^2^ indicate good model performance.

$$\text{RMSE}={\left(\left(\sum {\left(y\text{-}y_{\text{pred}}\right)}^{2}\right)/m\right)}^{0.5}$$ (10)

$${\text{R}}^{2}=1\text{-}\left(\sum {\left(y\text{-}y_{\text{pred}}\right)}^{2}\right)/\left(\sum {\left(y\text{-}y_{\text{mean}}\right)}^{2}\right)$$ (11)

where *y* is the actual value (observation), *y*_*pred*_ is the prediction, *m* is the number of the dataset sample, and *y*_mean_ is the mean value of the observations.

In addition to RMSE and R^2^, the a20-index, computed using Eq (12), is a recently proposed performance index and has been used in many studies [26, 133–135].

a20-index=m20/m (12)

where *m20* is the number of samples with a prediction-to-actual value ratio between 0.80 and 1.20. A high a20-index value indicates good model performance, with a value of 1 representing a perfect predictive model. The proposed a20-index has practical engineering significance, as it quantifies the proportion of samples where predictions fall within a ±20% deviation from the actual values.

### 2.8. Model for CO_2_ emissions

**Fig 3:**
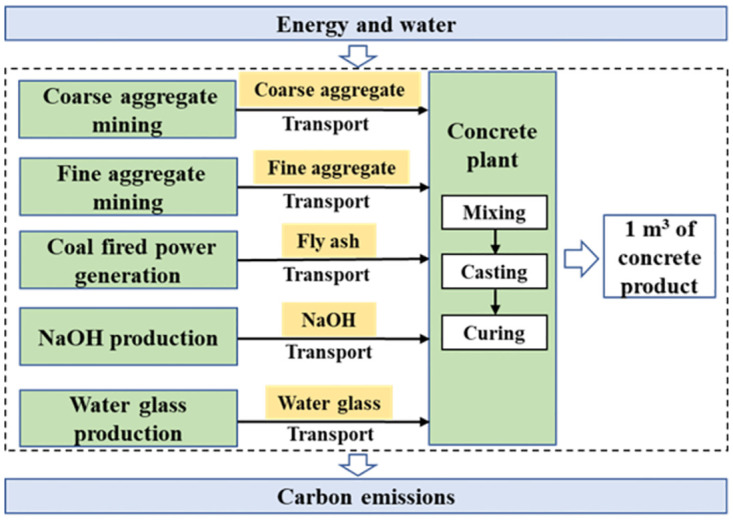
System boundary of CO_2_ emissions assessment.


Eq (13) gives a CO_2_ emissions model for 1 m^3^ FAGC [136–139] based on the life cycle assessment (LCA) from gate to cradle. The system boundary is shown in Fig 3. The CO_2_ emissions of concrete mixing and casting are assumed to be negligible.

$$\begin{array}{c} {\text{CO}}_{2}\text{emissions}=E_{RawMatProd}+E_{RawMatTrans}+E_{Curing}=\sum \left(F_{i}\times W_{i}\right)+\sum \left(F_{Trans}\times W_{i}\times D_{i}\right)+F_{Eng}\times {Eng}_{Curring} \end{array}$$ (13)

where *E*_*RawMatProd*_ is the CO_2_ emissions in the production of concrete raw materials (kgCO_2_/m^3^), *E*_*RawMatTrans*_ is the CO_2_ emissions in the transport of concrete raw materials (kgCO_2_/m^3^), *E*_*Curing*_ are the CO_2_ emissions in the concrete curing (kgCO_2_/m^3^), *F*_*i*_ is the *i*th concrete component’s CO_2_ emission factor (kgCO_2_/kg), *W*_*i*_ is the *i*th component’s weight in 1 m^3^ concrete (kg/m^3^), *F*_*Trans*_ is the CO_2_ emission factor of transport (kgCO_2_/kg-km), *D*_*i*_ is the *i*th component’s transport distance (km), *F*_*Eng*_ is the CO_2_ emission factor of the energy used, such as electricity, natural gas, solar energy, and so on (kgCO_2_/kWh), and *Eng*_*Curing*_ is the energy use of concrete curing (kWh).

Eq (14) is used for estimating the energy use of concrete curing [137, 139]. This equation is derived from the energy conservation. All the energy is utilized for heating the concrete to the curing temperature and offsetting heat loss.

$$Eng_{Curing}=Eng_{Heat}+Eng_{Loss}=M\times C\times \left(T\text{-}T_{0}\right)+P\times t_{1}/24$$ (14)

where *Eng*_*Heat*_ is the energy used for heating the concrete to the curing temperature, *Eng*_*Loss*_ is the energy used for offsetting heat loss, *M* is the concrete mass (kg), *c* is the specific heat capacity of FAGC, assumed to be 700 J/kg°C [137], *T* is the heating-curing temperature (°C), *T*_*0*_ is the ambient temperature (°C), *P* is the heating power for offsetting heat loss (kW), and *t*_*1*_ is the heat-curing time (day).

According to the heat transfer theory, the heating power for offsetting heat loss *P* is directly proportional to the temperature difference between *T* and *T*_*0*_ [139]. Obviously, *P* at *T*_*0*_ is 0. Defining *P* at *T* of 80°C as *P*_*0*_, Eq (15) is derived to calculate *P* at different curing temperatures.


$$P=\left(T\text{-}T_{0}\right)/\left(80^{{}^{\circ}}\text{C-}T_{0}\right)\times P_{0}$$ (15)

In summary, the CO_2_ emissions model, Eq (13), is actually a function of the mixture and curing parameters *x*, CO_2_ emissions = *f*(*x*).

### 2.9. Constraints for optimal mixture design

**Fig 4:**
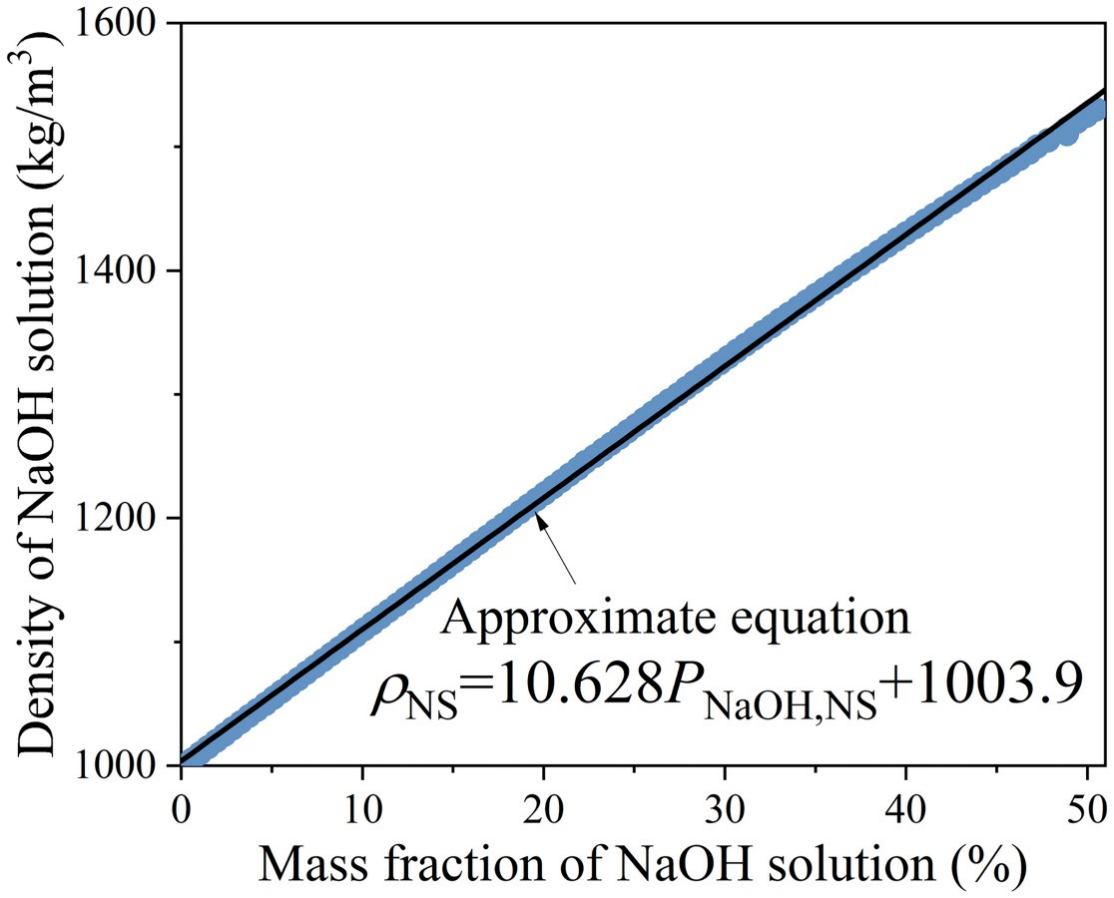
Relationship between density *ρ*_NS_ and mass fraction *P*_NaOH,NS_ of NaOH solution.

The optimal mixture design is determined by solving the optimization problem defined by the objective function and constraints. The objective function is to minimize CO_2_ emissions. The primary constraint is achieving the desired compressive strength, along with other constraints, as follows:

1) Compressive strength constraint: the compressive strength must not be less than the target compressive strength *f*_c,target_,
$$f_{\text{c},\text{pred}}(x)\geq f_{\text{c},\text{target}}$$ (16)
  where *f*_c,pred_(*x*) is the compressive strength prediction, which is actually a function of the mixture and curing parameters *x*.
2) Volume constraint: the volume fraction of each raw material and the air content adds up to one,
$$M_{\text{CoarseAgregate}}/\rho_{\text{C}}+M_{\text{FineAgeregate}}/\rho_{\text{F}}+M_{\text{FlyAsh}}/\rho_{\text{FA}}+M_{\text{Watergass}}/\rho_{\text{WG}}+M_{\text{NaOHSolution}}/\rho_{\text{NS}}+\alpha =1$$ (17)
  where *ρ*_i_ is the density of the *i*th raw material (kg/m^3^). α is the air content, which is usually assumed to 0.01. The density of NaOH solution *ρ*_NS_ varies with the mass fraction of NaOH solution *P*_NaOH,NS._ The relationship between *ρ*_NS_ and *P*_NaOH,NS_ is shown in Fig 4.
3) Workability constraint: *H*_*2*_*O/FA* must not be less than the required minimum value to ensure workability. Similarly, many studies reported that the fluidity sharply decreases when the aggregate volume increases to over 70% [117, 140]. Therefore, aggregate volume must not exceed 70% to ensure workability.
4) Non-negativity constraint: The mixture and curing parameters, as well as the mass fraction of NaOH solution, must not be less than 0.
5) Temperature and time constraints: The heat-curing temperature must be between 40°C and 120°C. And the heat-curing time must be between 1/6 and 4 days. These bounds are based on the observed range of values in the training set.

## 3. Results and discussion

### 3.1. Prediction models for compressive strength

**Fig 5:**
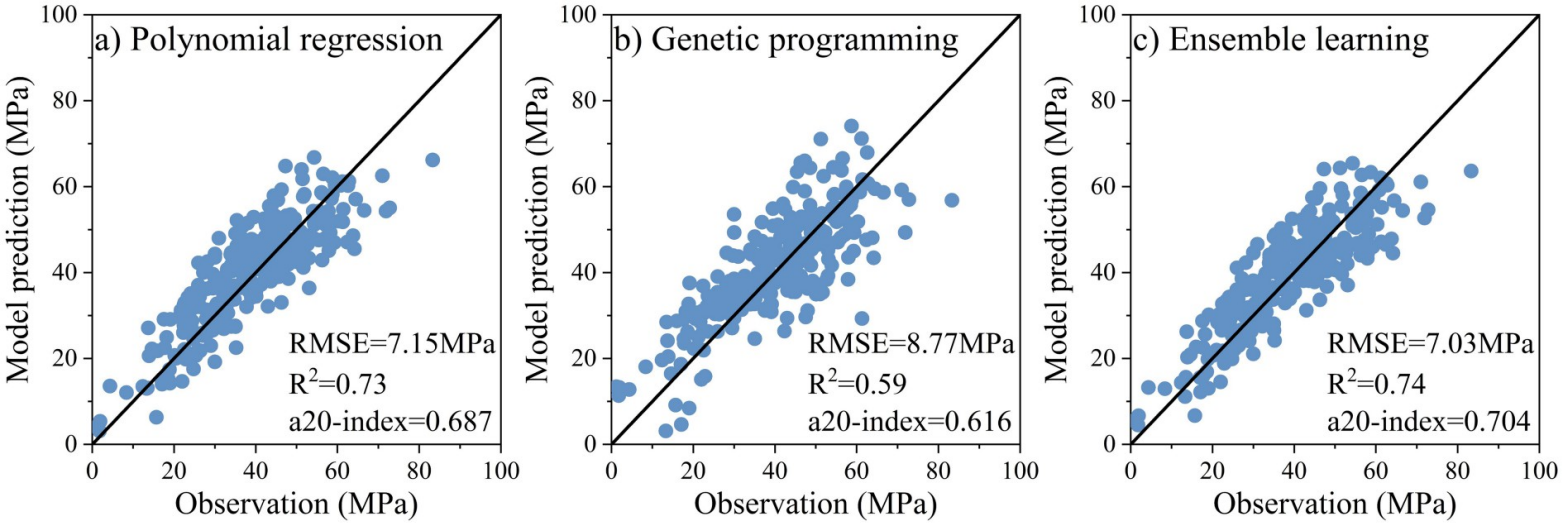
Comparison between the testing set’s observations and the three model’s predictions.

S1 Appendix shows the prediction models of the compressive strength of FAGC by polynomial regression, genetic programming, and ensemble learning, respectively. Fig 5 compares the testing set’s observations and the three models’ predictions. The ensemble learning model shows higher accuracy than polynomial regression model and genetic programming model in the testing set. The RMSE value decreases from 7.15 MPa to 7.03 MPa, the R^2^ value increases from 0.73 to 0.74, and the a20-index value increases from 0.687 to 0.704 after using ensemble learning model.

**Fig 6:**
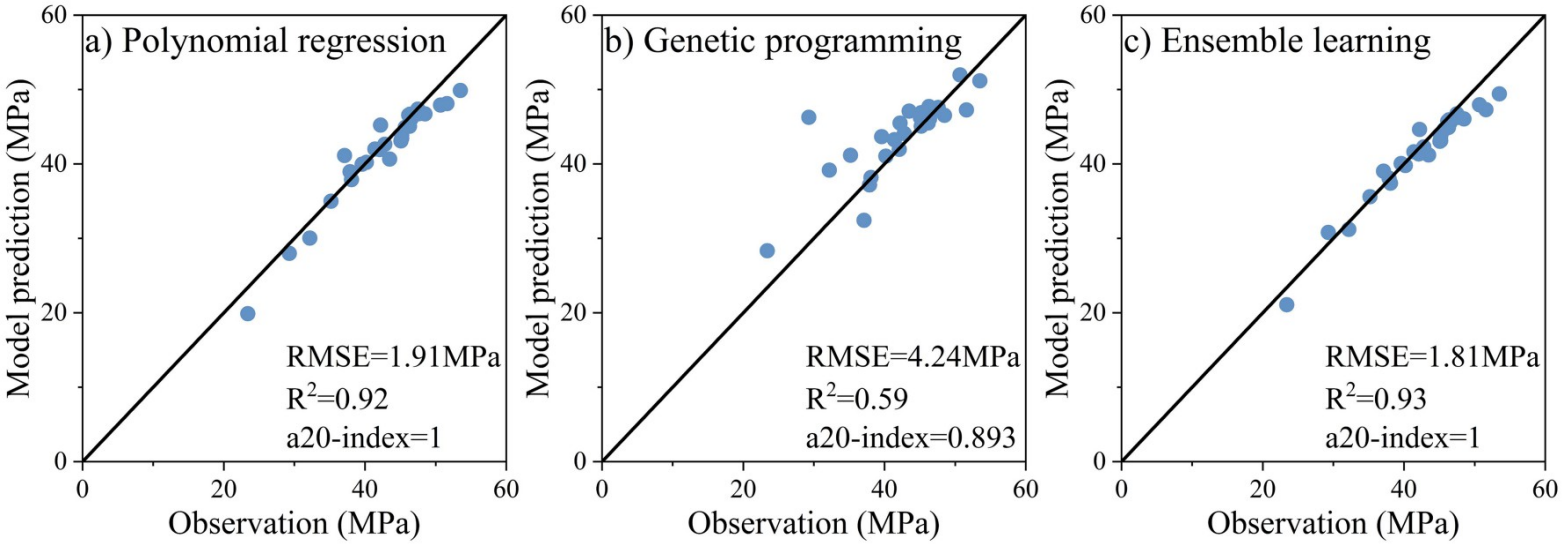
Comparison between the validation set’s observations and the three model’s predictions.

This study uses new experimental data to validate the prediction model of the compressive strength of FAGC. The experimental data (validation set) is given in S4 Table. Fig 6. compares the experimental results and the predicted results of the three models. The lowest RMSE value (1.81 MPa), the highest R^2^ value (0.93) and the highest a20-index value (1) indicate that the ensemble learning model successfully predicts the compressive strength of FAGC using a new fly ash. The ensemble learning model not only has a higher accuracy than the single model, but also shows better generalization ability to the new dataset.

Preventing overfitting in machine learning is essential for developing models that generalize well to new data. In this study, overfitting is expected to be mitigated by collecting more data and using an ensemble of multiple model predictions. Performance evaluations based on testing and experimental validation datasets demonstrate the success of these strategies in preventing overfitting. The resultant model demonstrates strong generalization to new data.

### 3.2. Effects of mix proportions and curing parameters on compressive strength

**Fig 7:**
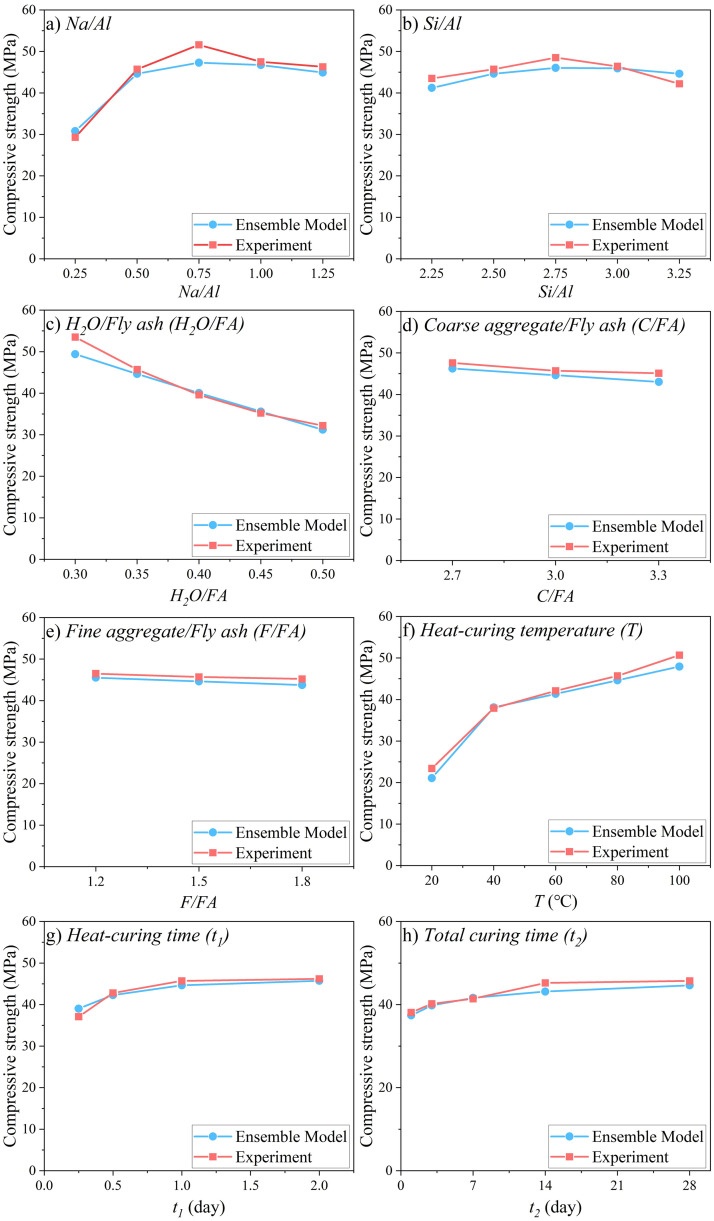
Effects of *Na/Al*, *Si/Al*, *H*_*2*_*O/FA*, *C/FA*, *F/FA*, *T*, *t*_*1*_, and *t*_*2*_ on compressive strength.

The effects of mix proportions and curing parameters on compressive strength of FAGC are shown in Fig 7, including both experimental data and model estimation. Although there are some deviations between the experimental data and model estimation, the overall trends remain consistent as follows:

1) Compressive strength initially increases with an increase in *Na/Al*, but then decreases with a further increase in *Na/Al*. In N-A-S-H, the positive charge of Na^+^ balances the negative charge resulting from the substitution of Al into a Q^4^ molecular coordination, and NaOH will be excess if Na/Al is greater than one where Al only refers to amorphous Al and does not include crystalline Al [141]. The excess NaOH is detrimental to N-A-S-H formation, and the strength decreases with the increase of *Na/Al* when *Na/Al* is greater than 1. The Al here is the whole Al, including amorphous Al and crystalline Al, and the actual optimal *Na/Al* value is less than 1.
2) Similarly, compressive strength initially increases with an increase in *Si/Al*, but decreases with a further increase. The saturation of Si in the reaction system due to increased Si from Na_2_SiO_3_ solution retards the dissolution of fly ash, resulting in minimal increase or even decrease in compressive strength. The optimal *Si/Al* is about 2.5 [142–144].
3) Compressive strength decreases linearly with an increase in *H*_*2*_*O/FA*. More water in the system creates more pores, leading to lower strength.
4) The content of coarse and fine aggregates has a minor impact on strength unless the amount of aggregate is excessive, preventing the paste from completely enveloping the aggregate and resulting in a porous concrete with significantly reduced strength.
5) Increasing the curing temperature enhances the compressive strength as it accelerates the reaction rate.
6) At high temperatures, e.g., 80°C, extending the curing time only marginally improves the compressive strength, resulting from the high reaction rate at high temperatures has made concrete to mature in a short period of time.

In summary, *Na/Al*, *H*_*2*_*O/FA*, and *T* are identified as key factors that obviously affect the compressive strength of FAGC.

### 3.3. Program for optimal mixture design

**Fig 8:**
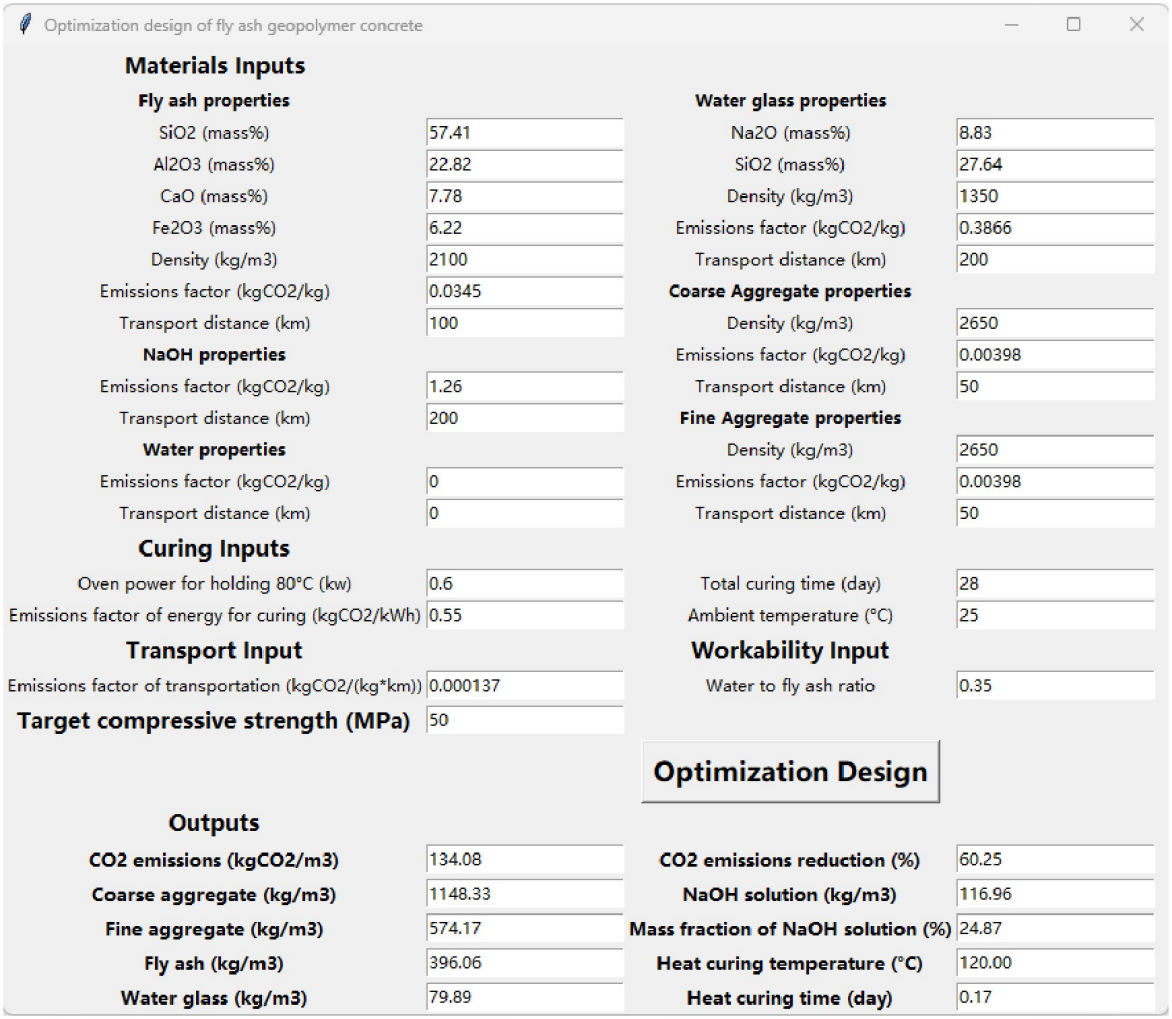
GUI of the program of the optimal FAGC mixture design.

The optimal mixture design method that satisfies target compressive strength and minimum CO_2_ emissions is developed based on the ensemble strength model and LCA-based CO_2_ emissions model. It is an optimization problem. The mixture and curing parameters *x* = (*M*_CoarseAggregate_, *M*_FineAggregate_, *M*_FlyAsh_, *M*_Waterglass_, *M*_NaOHSolution_, *P*_NaOH,NS_, *T*, *t*_*1*_), are the variables to be determined. These parameters represent the mass of each raw material, as well as the heating-curing temperature and time. The objective function is to minimize CO_2_ emissions, and achieving the desired compressive strength is one main constraint. Based on the objective function and constraints, the optimal mixture design program is implemented using the scipy.optimize.minimize algorithm within Python’s optimization toolbox. To facilitate the input of parameters, this study provides a graphical user interface (GUI), as depicted in Fig 8.

Once the user provides the necessary inputs, such as the parameters related to raw materials, curing, and transport, the required *H*_*2*_*O/FA*, and the desired target compressive strength, the program runs the optimization algorithm to find the optimal FAGC formula that minimizes CO_2_ emissions while satisfying the specified constraints.

The program outputs the optimal FAGC formula, including the mass fractions of each raw material and the curing parameters. Additionally, it calculates the carbon emissions associated with the optimal mixture design. Furthermore, the program provides information on the reduction in carbon emissions compared to an equivalent cement concrete with the same compressive strength and aggregate volume. The mixture design method of cement concrete follows the Chinese standard JGJ 55–2011 [145], where P·O 42.5 cement is used. P·O refers to ordinary Portland cement (OPC) that contains 80%-95% clinker and 5%-20% other materials such as slag or fly ash by mass, while the number 42.5 represents the strength class.

### 3.4. Case study for mixture design

Two cases about mixture design are presented, namely one using the optimal mixture design method developed in this study, and the other using the traditional trial and error design method. The latter involves creating multiple trial groups by adjusting the mixture and curing parameters (such as *Na/Al* and *T*) and testing them to find the one that achieves the target performance. The target compressive strength for both cases is set to 50 MPa, and the required parameters are shown in Fig 8.

Table 7 shows the design results. Both two mixtures can achieve the target strength. The traditional trial and error design result is from the experimental results as shown in S4 Table. Its CO_2_ emissions are 160.86 kgCO_2_/m^3^, which is calculated using Eq (13), while the carbon emissions of the FAGC mixture designed using the optimal mixture design method are only 134.08 kgCO_2_/m^3^, namely a decrease of 16.65%.

**Table 7:** Design results of FAGC.

	Trial and error design	Optimal mixture design
Coarse Aggregate (kg/m^3^)	1148.89	1148.33
Fine Aggregate (kg/m^3^)	574.44	574.17
Fly Ash (kg/m^3^)	382.96	396.06
Water glass (kg/m^3^)	134.50	79.89
NaOH (kg/m^3^)	36.08	29.12
Water in NaOH Solution (kg/m^3^)	48.59	87.84
Heat-curing temperature (°C)	80.00	120.00
Heat-curing time (day)	1.00	0.17
CO_2_ emissions (kgCO_2_/m^3^)	160.86	134.08
Compressive strength (MPa)	51.60	50.70

Comparing the two design methods, the optimal mixture design method exhibits lower water glass content and shorter heat-curing time but a higher heat-curing temperature compared to the trial and error method. This is consistent with the explanation of the model in Section 3.2. It is observed that when the Si content is already high, further increasing it has little effect on strength improvement. Additionally, at high temperatures, extending the curing time only marginally improves the compressive strength, while increasing the heat-curing temperature significantly enhances the compressive strength. The optimization algorithm chooses to reduce the water glass content and heat-curing time while increasing the heat-curing temperature to effectively compensate for the strength loss in a cost-effective manner.

Furthermore, this study compares the CO_2_ emissions of the optimal FAGC with equivalent cement concrete having the same compressive strength and aggregate volume. The CO_2_ emissions of the cement concrete are 337.29 kgCO_2_/m^3^, while the optimal FAGC emits only 134.08 kgCO_2_/m^3^, achieving a significant reduction of 60.25%. This highlights the potential of FAGC as a low-carbon alternative to cement concrete.

## 4. Conclusions

This study focuses on computational mixture design optimization to develop FAGC that meets key performance targets, such as compressive strength, while minimizing CO_2_ emissions. First, a robust model for predicting the compressive strength of FAGC is developed. Comprehensive factors, including fly ash characteristics, mixture proportions, curing parameters, and specimen types, are considered. A big dataset comprising 1136 observations is established. Polynomial regression, genetic programming, and ensemble learning techniques are employed. Subsequently, using the developed strength model and a life cycle assessment (LCA)-based CO_2_ emissions model, an optimal FAGC mixture design program is formulated. The key findings are as follows:

- The ensemble learning model exhibits superior accuracy and generalization ability compared to single models for compressive strength prediction of FAGC, achieving the lowest RMSE value of 1.91 MPa, the highest R^2^ value of 0.93, and the highest a20-index value of 1.
- The experimental data and the ensemble learning model identify Na to Al molar ratio (*Na/Al*), water to fly ash mass ratio (*H*_*2*_*O/FA*), and curing temperature (*T*) as the key factors influencing the compressive strength of FAGC.
- In the FAGC mixture design case, the optimal design, as opposed to traditional trial-and-error methods, leads to a 16.7% reduction in CO_2_ emissions for FAGC with a compressive strength of 50 MPa. Moreover, compared to conventional cement concrete, the developed FAGC demonstrates a substantial 61% reduction in CO_2_ emissions.
- The results of this work provide engineers with tools for compressive strength prediction and low-carbon optimization of FAGC, enabling rapid and highly accurate design of concrete with lower CO_2_ emissions and greater sustainability.

## 5. Limitations and future work

In modeling the compressive strength of FAGC, this study successfully employs an ensemble machine learning model that demonstrates superior performance compared to single models. However, there is room for improvement in the proposed approach. Future enhancements could include integrating an artificial neural network as a third base learner in the ensemble, alongside polynomial regression and genetic programming models, and using genetic programming or artificial neural network instead of linear regression to aggregate base learners and create the ensemble model. In addition, although this work considers a more comprehensive set of input variables than previous work, some influential factors remain unaccounted for, particularly fly ash characteristics such as amorphous content, fineness, and density. This is because most studies do not report this information. Future experiments that provide this information could lead to the inclusion of additional input parameters, thereby improving prediction accuracy.

Regarding mixture design optimization, this study focuses on developing FAGC that meets target compressive strength while minimizing CO_2_ emissions. Building on the ensemble modeling method proposed in this work, and using the database of workability and durability, future work could develop prediction models for these additional performance metrics. These models could then be integrated into the optimization framework, enabling multi-performance optimization, e.g., meeting target compressive strength, workability, and durability while minimizing CO_2_ emissions.

## Supporting information

S1 Appendix Compressive strength prediction models of FAGC. (DOCX)

S1 Table Compressive strength of FAGC and related information. (XLSX)

S2 Table Training set. (XLSX)

S3 Table Testing set. (XLSX)

S4 Table Validation set. (XLSX)

## Abbreviations

*c*: Specific Heat Capacity
*C/FA*: Coarse Aggregate to Fly Ash Mass Ratio
*D*_*i*_: *i*th Concrete Component’s Transport Distance
*E*_*Curing*_: CO_2_ Emissions from Concrete Curing
*Eng*_*Curing*_: Energy Use of Concrete Curing
*Eng*_*Heat*_: Energy Use for Heating
*Eng*_*Loss*_: Energy Use for Offsetting Heat Loss
*E*_*RawMatProd*_: CO_2_ Emissions from Production of Concrete Raw Materials
*E*_*RawMatTrans*_: CO_2_ Emissions from Transport of Concrete Raw Materials
*F/FA*: Fine Aggregate to Fly Ash Mass Ratio
FAGC: Fly Ash Geopolymer Concrete
*f*_c,pred_(*x*): Compressive Strength Prediction
*f*_c,target_: Target Compressive Strength
*F*_*Eng*_: CO_2_ Emission Factor of Energy
*F*_*i*_: *i*th Concrete Component’s CO_2_ Emission Factor
*F*_*Trans*_: CO_2_ Emission Factor of Transport
*H*_*2*_ *O/FA*: Water to Fly Ash Mass Ratio
LCA: Life Cycle Assessment
*M*: Concrete Mass
*m*: Number of Dataset Sample
*m20*: Number of Samples with Prediction-to-Actual Value Ratio between 0.80 and 1.20
*M*_CoarseAggregate_: Coarse Aggregate Mass in 1 m^3^ FAGC
*M*_FineAggregate_: Fine Aggregate Mass in 1 m^3^ FAGC
*M*_FlyAsh_: Fly Ash Mass in 1 m^3^ FAGC
*M*_H2O,NS_: Water Mass in NaOH Solution
*M*_H2O,WG_: Water Mass in Water Glass
*M*_NaOH_: NaOH Mass in 1 m^3^ FAGC
*M*_NaOHSolution_: NaOH Solution Mass in 1 m^3^ FAGC
*M*_Waterglass_: Water Glass Mass in 1 m^3^ FAGC
*Na/Al*: Sodium to Aluminum Molar Ratio
P: Heating Power for Offsetting Heat Loss
*P*: Heating Power for Offsetting Heat Loss at Temperature of 80°C
*P*_Al2O3,FA_: Mass percent of Al_2_O_3_ in Fly Ash
*P*_CaO,FA_: Mass percent of CaO in Fly Ash
PCC: Portland Cement Concrete
*P*_Fe2O3,FA_: Mass percent of Fe_2_O_3_ in Fly Ash
*P*_Na2O,WG_: Mass percent of Na_2_O in Water Glass
*P*_NaOH,NS_: Mass percent of NaOH in NaOH Solution
*P*_SiO2,FA_: Mass percent of SiO_2_ in Fly Ash
*P*_SiO2,WG_: Mass percent of SiO_2_ in Water Glass
R^2^: Coefficient of Determination
RMSE: Root Mean Squared Error
*Si/Al*: Silicon to Aluminum Molar Ratio
*T*: Curing Temperature
*T*_*0*_: Ambient Temperature
*t*_*1*_: Heat-Curing Time
*t*_*2*_: Total Curing Time
*W*_*i*_: *i*th Component’s Weight in 1 m^3^ Concrete
*y*: Actual Value (Observation) of Sample
*y*_mean_: Mean Value of Observations
*y*_*pred*_: Prediction of Sample
α: Air Content
*ρ*_C_: Density of Coarse Aggregate
*ρ*_F_: Density of Fine Aggregate
*ρ*_FA_: Density of Fly Ash
*ρ*_NS_: Density of NaOH Solution
*ρ*_WG_: Density of Water Glass
