# Expression of Concern: Compressive strength prediction and low-carbon optimization of fly ash geopolymer concrete based on big data and ensemble learning

**DOI:** 10.1371/journal.pone.0346735

**Published:** 2026-04-08

**Authors:** The PLOS One Editors

After this article [1] was published, the *PLOS One* Editors identified concerns about the article’s peer review. We regret that the issues were not addressed prior to the article’s publication. Readers are advised to interpret the article [1] with caution.
