# Supplementary material for: Compressive strength prediction and low-carbon optimization of fly ash geopolymer concrete based on big data and ensemble learning
Source: PLoS One. 2024 Sep 12;19(9):e0310422. doi: 10.1371/journal.pone.0310422 (PMC11392388; doi:10.1371/journal.pone.0310422)
Supplement: S1 Appendix — (DOCX) [file pone.0310422.s001.docx]

# **Compressive Strength Prediction and Low-carbon optimization of Fly Ash Geopolymer Concrete Based on Big Data and Ensemble Learning**

Peiling Jiang ^1^, Diansheng Zhao ^2^, Cheng Jin ^3^, Shan Ye ^1^, Chenchen Luan ^4,*^, Rana Faisal Tufail ^5^

^1^ Zhejiang Tongji Vocational College of Science and Technology, Zhejiang, China

^2^ Zhejiang University of Technology, Zhejiang, China

^3^ Zhejiang University of Technology Engineering Design Group Co. Ltd, Zhejiang, China

^4^ School of Civil and Environmental Engineering, Harbin Institute of Technology, Shenzhen, China

^5^ Civil Engineering Department, Wah Campus, COMSATS University Islamabad, Rawalpindi 45550, Pakistan

* Corresponding author

E-mail: [luanchenchen@hit.edu.cn](mailto:luanchenchen@hit.edu.cn) (CL)

**Polynomial regression:** *f_c_=f(x)=161.131-259.195*x2-133.148*x6-38.193*x7+18.036*x10-406.192*x0*x0-299.829*x1*x1+70.035*x2*x2-93.075*x4*x4-687.644*x5*x5-1.95*x10*x10+425.234*x0*x2-621.438*x0*x3-177.928*x0*x4+890.9*x0*x5+123.223*x0*x9-147.145*x1*x2+602.645*x1*x3-529.344*x1*x5+88.853*x1*x7+250.144*x1*x11+41.834*x2*x6+383.683*x3*x5+296.852*x4*x5+92.827*x4*x11+42.466*x5*x7-22.29*x6*x7+50.517*x6*x8-6.43*x7*x9-40.319*x8*x11-17.564*x9*x10-35.796*x9*x11* (S1)

**Genetic programming:** *f_c_=g(x)=x0/0.026+(x2+x11+x11-x9-(x5-(x11+x3)-(x9/0.193+x3-0.946+x2-(x11/x10-(x3-0.946+x11)-(x2-x11/x10+(x3-0.946+x11))))))*(x3/x6+x2/x6/0.491-((x0-x2/x10/0.491)*(x9/0.193+x6/x2-x1)-(x2/x6/0.491-(x5-x10)*(-0.946-x7)))*(-0.065+x2-x5*x5)*((x0-x7)*(x2-x1*x4)+(x0+x3+x5-x6)+x5))* (S2)

**Ensemble learning:** *f_c_=0.811*f(x)+0.175*g(x)* (S3)
